# A Swarm of Quacks; Medical College of Ohio; Old Times

**Published:** 1884-07

**Authors:** 


A SWARM OF QUACKS.—It is said that recent legislation in neighboring States has driven so many quacks into Iowa that the regular profession are seriously considering the ways and means by which these pests may be legally driven forth to forage in distant States.

MEDICAL COLLEGE OF OHIO.—The faculty of the Medical College of Ohio has decided to hold a preliminary examination of all candidates for admission to the lecture classes, in the absence of a diploma or other evidence of sufficient literary training. A chair of Hygiene has also been created.

OLD TIMES.—From a French state paper, lately brought to light, it appears that in 1770 the following parliamentary decree was solemnly passed and duly registered under King Louis XV: "Whosoever, by means of red or white paint, perfumes, essences, artificial teeth, false hair, cotton, wool, iron corsets, hoops, shoes with high heels, or false hips, shall seek to entice into the bands of marriage any male subject of His Majesty, shall be prosecuted for witchcraft, and declared incapable of matrimony."

